# Genome Editing of *Wnt-1*, a Gene Associated with Segmentation, via CRISPR/Cas9 in the Pine Caterpillar Moth, *Dendrolimus punctatus*

**DOI:** 10.3389/fphys.2016.00666

**Published:** 2017-01-06

**Authors:** Huihui Liu, Qun Liu, Xuguo Zhou, Yongping Huang, Zhen Zhang

**Affiliations:** ^1^Key Laboratory of Forest Protection, Research Institute of Forest Ecology, Environment and Protection, Chinese Academy of Forestry, State Forestry AdministrationBeijing, China; ^2^Key Laboratory of Insect Developmental and Evolutionary Biology, Institute of Plant Physiology and Ecology, Shanghai Institutes for Biological Sciences, Chinese Academy of SciencesShanghai, China; ^3^Department of Entomology, University of KentuckyLexington, KY, USA

**Keywords:** *Dendrolimus punctatus*, CRISPR/Cas9, *Wnt-1*, segmentation, embryogenesis, genome editing

## Abstract

The pine caterpillar moth, *Dendrolimus punctatus*, is a devastating forest pest. Genetic manipulation of this insect pest is limited due to the lack of genomic and functional genomic toolsets. Recently, CRISPR/Cas9 technology has been demonstrated to be a promising approach to modify the genome. To investigate gene functions during the embryogenesis, we introduced CRISPR/Cas9 system in *D. punctatus* to precisely and effectively manipulate gene expressions inmutant embryos. Compared to controls, knocking out of *DpWnt-1*, a gene well known for its role in the early body planning, led to high embryonic mortality. Among these mutants, 32.9% of the embryos and larvae showed an abnormal development. *DpWnt-1* mutants predominantly exhibited abnormal posterior segments. In addition, multiple phenotypes were observed, including the loss of limbs and the head deformation, suggesting that *DpWnt-1* signaling pathway is necessary for anterior segmentation and appendage development. Overall, our results demonstrate that CRISPR/Cas9 system is feasible and efficient in inducing mutations at a specific locus in *D. punctatus*. This study not only lays the foundation for characterizing gene functions in a non-model species, but also facilitates the future development of pest control alternatives for a major defoliator.

## Introduction

The pine caterpillar moth *Dendrolimus punctatus* (Lepidoptera: Lasiocampidae) is one of the most destructive forest pests in China and Southeast Asia, where it attacks a variety of pine species and causes extensive forest damages (Billings, 1991; Zeng et al., 2010). Through the years, *D. punctatus* management has relied primarily on synthetic insecticides. The advent of Genomic Era facilitates the development of environmentally friendly and sustainable control alternatives. The sterile insect technique (SIT) is an environmentally friendly insect control technology that relies on the release of large numbers of sterile males to mate with wild females to suppress pest population (Benedict and Robinson, 2003). The application of this method, however, is limited by the production of undesired females which need to be separated and eliminated. A modified SIT technique, the release of insects carrying a conditional dominant lethal gene (RIDL) can overcome this issue by inducing repressible female-specific lethality (Heinrich and Scott, 2000; Horn and Wimmer, 2003; Fu et al., 2007; Windbichler et al., 2008; Tan A. et al., 2013). This concept has been proofed in the mosquito control, both in laboratory and confined field tests (Thomas et al., 2000; Alphey and Andreasen, 2002; Alphey et al., 2002).

RNAi and genome editing are the primary tools to elucidate gene functions (Mao et al., 2013; Ma et al., 2014; Xu et al., 2014, 2015; Hammond et al., 2016). However, RNAi efficiency is highly variable in lepidopterans which underlying mechanisms are still unknown. More importantly, heritable RNAi effects have yet to be documented in lepidopterans (Bettencourt et al., 2002; Terenius et al., 2011; Swevers and Smagghe, 2012). In contrast, genome editing can achieve target gene mutagenesis by inducing irreversible DNA breaks (Corrigan-Curay et al., 2015). Genome editing tools, including customized zinc-finger nucleases (ZFN), transcription activator-like effector nucleases (TALEN) or clustered regularly interspaced short palindromic repeats-associated nuclease 9 (CRISPR-Cas9), can effectively modify the genomic DNA of organisms. By inducing DNA double-stranded breaks (DSBs), these tools stimulate subsequent homologous recombination (HR) and/or non-homologous ends-joining (NHEJ), which facilitate genome manipulation at a target locus (Harrison et al., 2014). Although ZFN and TALEN have been used for gene targeting, the complexity of module construction and the costs associated with these tools limit their applications. Recently, a bacteria-derived CRISPR/Cas9 system, consisting of CRISPR RNAs and Cas proteins, circumvents some of these issues. With the aid of two short RNA molecules, namely CRISPR RNA (crRNA) and trans-encoded CRISPR RNA (tracrRNA), the Cas9 endonuclease can cleave a specific sequence that is targeted by the RNAs. These two RNA molecules can be fused artificially to form a chimeric RNA molecule called single guide RNA (sgRNA). CRISPR/Cas9 system has been used to produce heritable mutations in non-model organisms, including RNAi-recalcitrant Lepidoptera, such as *Bombyx mori, Danaus plexippus, Spodoptera litura, Plutella xylostella, Spodoptera littoralis*, and *Helicoverpa armigera* (Wang et al., 2013, 2016; Daimon et al., 2014; Huang et al., 2016; Koutroumpa et al., 2016; Markert et al., 2016; Zhu et al., 2016).

To facilitate the construction of RIDL, we focus on the search of targeting genes, including lethal genes. In *Drosophila, wingless* also called *Wnt Family Member 1* (*Wnt-1*), is associated with wing development (Sharma and Chopra, 1976). *Wnt/*β*-catenin* signalingis highly conserved in insects, can control cell fate and proliferation, and determine body plan in vertebrate embryos (Hikasa and Sokol, 2013). While *Wnt/*β*-catenin* signaling is required for segmentation during the early embryogenesis (Bolognesi et al., 2008; Petersen and Reddien, 2009; Fu et al., 2012), it also involves in the renewal of epithelial tissue (Sahai-Hernandez et al., 2012), antero-posterior brain patterning (Kobayashi et al., 2007), long-term memory (Tan Y. et al., 2013), neural plate and planarian regeneration (Niehrs, 2010) and head formation (Posnien et al., 2010). In *Tribolium, Wnt* signaling plays important roles in leg development during the embryonic stage, also involves in leg and wing regeneration, and in metamorphosis (Ober and Jockusch, 2006; Shah et al., 2011). In Lepidoptera, including *Manduca sexta* and *B. mori, Wnt-1* contributes to the posterior growth and segmentation processes (Kraft and Jäckle, 1994; Zhang et al., 2015). In other species of vertebrate and invertebrate, *Wnt*-signaling genes are involved in the head morphogenesis and appendage development (Heisenberg et al., 2001; Müller et al., 2007; Lewis et al., 2008; Eroshkin et al., 2016).

The segmentation process involves multiple genes' interactions. In *Drosophila, Wnt* suppressed *hedgehog* (*hh*) and *engrailed* (*en*) expression in intercalary stripe and antennal stripe, but initiated *en* expression in ocular segment (Gallitano-Mendel and Finkelstein, 1997). A cephalic gap genes *Orthodenticle (otd)* represses *wg* expression in the antennal segment and all segments posterior to it, but activates *wg* expression in ocular segment (Gallitano-Mendel and Finkelstein, 1998). In *Tribolium*, complementary cross-regulation of *Wnt* and *Hh* pathways play an opposite interaction in the head and trunk development (Oberhofer et al., 2014). Knockout of *Axin*, a negative regulator of the *Wnt* pathway, led to missing head and thorax (Fu et al., 2012). A similar phenotype was obtained from *Masterblind*/*Axin1* mutation, which showed smaller head and eyes in zebrafish (Heisenberg et al., 2001). In *Xenopus laevis, Noggin4* regulates head development by inhibiting *Wnt8* signaling (Eroshkin et al., 2016). In mouse, DKK (*Dickkopf* -related protein 1) as one of *Wnt* antagonists, is expressed anteriorly to repress *Wnt* signaling in the head (Lewis et al., 2008). In *Hydractinia*, activation of *Wnt* signaling by blocking *GSK-3*β(*Glycogen Synthase Kinase 3*β) affected regeneration, the patterning of growing polyps and the asexual formation of new polyps in the colony (Müller et al., 2007).

In this study, we explored CRISPR/Cas9-based genome editing in a major forest pest in China, the pine caterpillar moth, *D. punctatus*. Our molecular target, *Wnt-1*, is believed to be involved in the body plan in *D. punctatus*. To test this functional genomics tool, we first cloned the *DpWnt-1*, and then generated loss-of-function mutations through microinjection at the embryonic stage. The resultant phenotypic impacts of *Wnt-1* knockout included lethality, abnormal segmentation and defective appendages. This proof-of-concept study using the CRISPR/Cas9-based genome editing tool demonstrates the feasibility of the genetic manipulation in a forest insect pest, which bears promising future advances in functional genomic research in forest entomology.

## Materials and methods

### Gene identification, motif, and phylogenetic analyses

To search for the *Wnt-1* homolog, nucleotide sequence of *BmWnt-1* (NM_001043850.1) was used as a query to BLAST against a *D. punctatus* transcriptome (HHL, unpublished data). RACE was used to obtain the full length cDNA of *DpWnt-1*. The predicted open reading frame (ORF) was subjected to motif search, pattern analysis, and phylogenetic analysis. The MEME online server was used for motif analysis, and parameters were as follows: a minimum width was 6; a maximum width was 12; and a maximum number of motif was 8 (http://meme-suite.org/tools/meme). To understand the phylogenetic relationship of *DpWnt-1* with homologs from other animals, a neighbor-joining tree was constructed using MEGA5, http://www.mega-software.net/ (Tamura et al., 2011). The *Wnt-1* ORFs included in the analysis are as follows: *B*. *mori* (NM_001043850), *H. armigera* (KJ206240), *Amyelois transitella* (XM_013345048), *P. xylostella* (XM_011569928), *M. sexta* (Z30280), *P. xuthus* (XM_013325799), *Danio rerio* (XP_005162280), *Fopius arisanus* (XM_011300877), *Bombus terrestris* (XM_003393116), *Nasonia vitripennis* (XM_001603338), *Bactrocera dorsalis* (XM_011204079), *Drosophila willistoni* (XM_002066877), *Drosophila melanogaster* (NM_078778), *Tribolium castaneum* (EFA04660), *Periplaneta americana* (KC311252), *Gryllus bimaculatus* (BAB19660), *Homo sapiens* (NP_005421) and *Mus musculus* (NP_067254).

### cDNA cloning and sequence analysis

Total RNA was isolated with Trizol Reagent (Invitrogen, USA) from *D. punctatus* pupae. Recombinant DNase I-treated (Takara, Japan) RNA was used for cDNA synthesis with the Scientific Revert Aid First Strand cDNA synthesis kit (Thermo, USA). Diluted reverse transcription products were used as templates to amplify DNA fragments. The primer sets used to obtain the *DpWnt-1* ORF are listed in Table 1. Template DNA was denatured at 94°C for 2 min, followed by 35 cycles of 94°C for 15 s, 55°C for 30 s and 68°C for 1.5 min. PCR products were cloned into the pCR-Blunt vector for sequencing by ABI 3730 XL sequencer (Applied. Biosystems, USA).

**Table 1:** **Primers used in this study**.

**Name**	**Sequence(5′–3′)**	**Purpose**
*Wnt1-sgRNA-a*	TAATACGACTCACTATAGGATGAGGTTACCTAGCTTTGTTTTAGAGCTAGAAATAGCAAGTTAAAA	sgRNA
*Wnt1-sgRNA-b*	TAATACGACTCACTATAGGTGTCTCTAAATCCACGTTGTTTTAGAGCTAGAAATAGCAAGTTAAAA	
*EGFP-sgRNA-a*	TAATACGACTCACTATAGGGCGAGGAGCTGTTCACCGGTTTTAGAGCTAGAAATAGCAAGTTAAAA	
*EGFP-sgRNA-b*	TAATACGACTCACTATAGGCCACAAGTTCAGCGTGTCGTTTTAGAGCTAGAAATAGCAAGTTAAAA	
*sgRNA-R*	AAAAGCACCGACTCGGTGCCACTTTTTCAAGTTGATAACGGACTAGCCTTATTTTAACTTGCTATT	
*Wnt1-ORF-F*	CCGCCCATCCCAGAATGAAGTGTC	ORF
*Wnt1-ORF-R*	CTATAAGCACGTATGCACCACTT	
*Wnt1-Test-F*	CACGTGCAAACGGAGATGCGGCA	Somatic mutation
*Wnt1-Test-R*	CTATAAGCACGTATGCACCACTT	
*Wnt-1-F*	TGTCCGTGGTTGTTTGTGTT	qRT-PCR
*Wnt-1-R*	TATTTGGTTCTCCCGCTTTG	
*Abd-a-F*	GGGAGGAGCAGGAGAGAATG	
*Abd-a-R*	CTTTGAGTAGGTCGTTGGA	
*Ubx-F*	ATTTTGAGCAGGGTGGCTTT	
*Ubx-R*	GAGGCTGGGCATAGGTGAG	
*Abd-b-F*	GTGGCGAAGAACGGCGGACA	
*Abd-b-R*	GAAGAACCGCAGCCGACCCC	
*Scr-F*	GTAGAGCAAACGGGGCATC	
*Scr-R*	TGCGGTGGCGAGTAACAA	
*Antp-F*	CGTATGAAGTGGAAGAAGGAGAA	
*Antp-R*	TATTGTGGCGAGGTTGGTG	
*Dfd-F*	GCTGGAGTCACCACCACGGC	
*Dfd-R*	TGCCCACCGACGCAATGCAA	
*Lab-F*	GATACCGCCCGCAGAGTT	
*Lab-R*	TGTTGTTGAGATTTAGGAGTGG	
*Pb-F*	AGTGGAACGCAAAACACAAA	
*Pb-R*	GAAGTGGAAGTCTGAGGAGGAG	
*RP32-F*	ATGGCAATCAGACCTGTGTACAG	
*RP32-R*	GACGGGTCTTCTTGTTTGATCCGT	

### Quantitative real-time PCR (qRT-PCR) analysis

qRT-PCR was performed to analyse the expression profile of *DpWnt-1* and 8 *Hox* genes during the embryonic stage. cDNA samples were prepared from embryos of different developmental stages (day 1–day 8 of wild type) and the first instar larvae of *DpWnt-1* mutants. Mastercycler EP realplex (Eppendorf) was used for the qRT-PCR. The primer sets used in qRT-PCR analysis are listed in Table 1. The cycling conditions were as follows: an initial incubation at 95°C for 10 s, 40 cycles of 95°C for 15 s, and 60°C for 30 s according to SYBR Green fluorescent relative quantitative approaches (TaKaRa, Japan). The relative mRNA level of the target genes was calculated using the 2^−ΔΔCt^ method, in which the target gene expression was normalized to an internal reference, *RP32*. Three independent replications for each sample were performed.

### *In vitro* transcription of Cas9 and sgRNA

The Cas9 gene template used in this work was provided by View Solid Biotech (Beijing, China). Cas9 mRNA was synthesized *in vitro* with the mMESSAGE mMACHINE® T7 kit (Ambion, USA) according to the manufacturer's instructions.

For the *in vitro* transcription of sgRNA driven by the T7 promoter, target sequences start with GG. With the PAM sequences in consideration, the designed sgRNA sites follow the GGN_19_GG rule (Wang et al., 2013). We identified two 23 bp sgRNA targeting sites at exon III of *DpWnt-1* (**Figure 3A**). The control sgRNAs were used for targeting the EGFP gene. Two complementary oligonucleotides were annealed and cloned into pJET1.2 (Fermentas, USA). The templates for *in vitro* transcription were amplified from pJET1.2, and primer sets used in this study are listed in Table 1. sgRNAs were transcribed *in vitro* with the MAXIscript® T7 kit (Ambion, USA), following the manufacturer's recommended protocol.

### Colony maintenance and embryonic microinjection

*Dendrolimus punctatus* pupae were originally obtained from Xing'an County of Guilin city, Guangxi province, P.R. China. *D. punctatus* colonies were provisioned with Masson's pine, and maintained at 27 ± 1°C under a L/D cycle of 16/8 h. Fertilized eggs were collected within 2 h after oviposition, and subjected to microinjection.

The combination of Cas9 mRNA (300 ng/μl) and sgRNAs (sgRNA-a and sgRNA-b, 300 ng/μl, respectively), and Cas9 mRNA/sgRNAs (sgRNA-a and sgRNA-b) (500 ng/μl each) were co-injected into preblastoderm embryos. An exogenous gene EGFP and nuclear free water without any sgRNAs or Cas9 mRNA were used as control. These control should have none effect on the embryonic development. Injection was carried out following Tamura et al. (1990) with modification, and injection site was shown in Figure S2. As the egg is oval in shape, we lined up the egg with the micropyle on top and injected compounds to the gonad region. The microinjection was concluded within 6 h. Afterwards, the injected eggs were incubated at 25 ± 1°C in a humidified chamber for 8–10 days until hatch. All hatched larvae were collected and transferred to Masson's pine.

### Phenotype documentation and mutation screening

The injected embryos were dissected and checked to calculate the mutation rate and hatching rate on the seventh day of the embryonic stage, and the resultant phenotypes were documented under a multi-function zoom microscope (AZ100, Nikon). The images were recorded with a computer-controlled microscope system. The pictures of *DpWnt-1* mutants, including both larvae and pupae, were taken by SLR cameras.

To calculate the efficiency of Cas9/sgRNA-mediated gene alteration in the injected generation, individuals were collected on the eighth day after injection. The DNA fragments surrounding the sgRNA targets were obtained by GBdirect PCR directly from embryos (GBI, China). The primer sets are shown in Table 1. Mutations were confirmed by sequencing.

### Immunoblotting analysis

Proteins from 7 day old embryos were used for the immunoblotting analysis. The primary antibodies, *B. mori Anti-Wnt-1* and *Anti-*β*-actin*, respectively, were used at 1:1000 dilution. The secondary antibody, anti-rabbit IgG, was diluted at 1:5000. Proteins were extracted and diluted with PBS and quantified using bicinchoninic acid (BCA) protein assay kit (Thermo). A 12.5% SDS-PAGE gel was used to separate the same amount of proteins from both the wild types and mutants. The proteins were then transferred to a polyvinylidene fluoride membrane. Signal visualization was obtained using the ECL Plus Western Blotting detection kit (GE Health-care).

## Results

### Expression profile of *DpWnt-1* during embryogenesis

**Figure 1:**
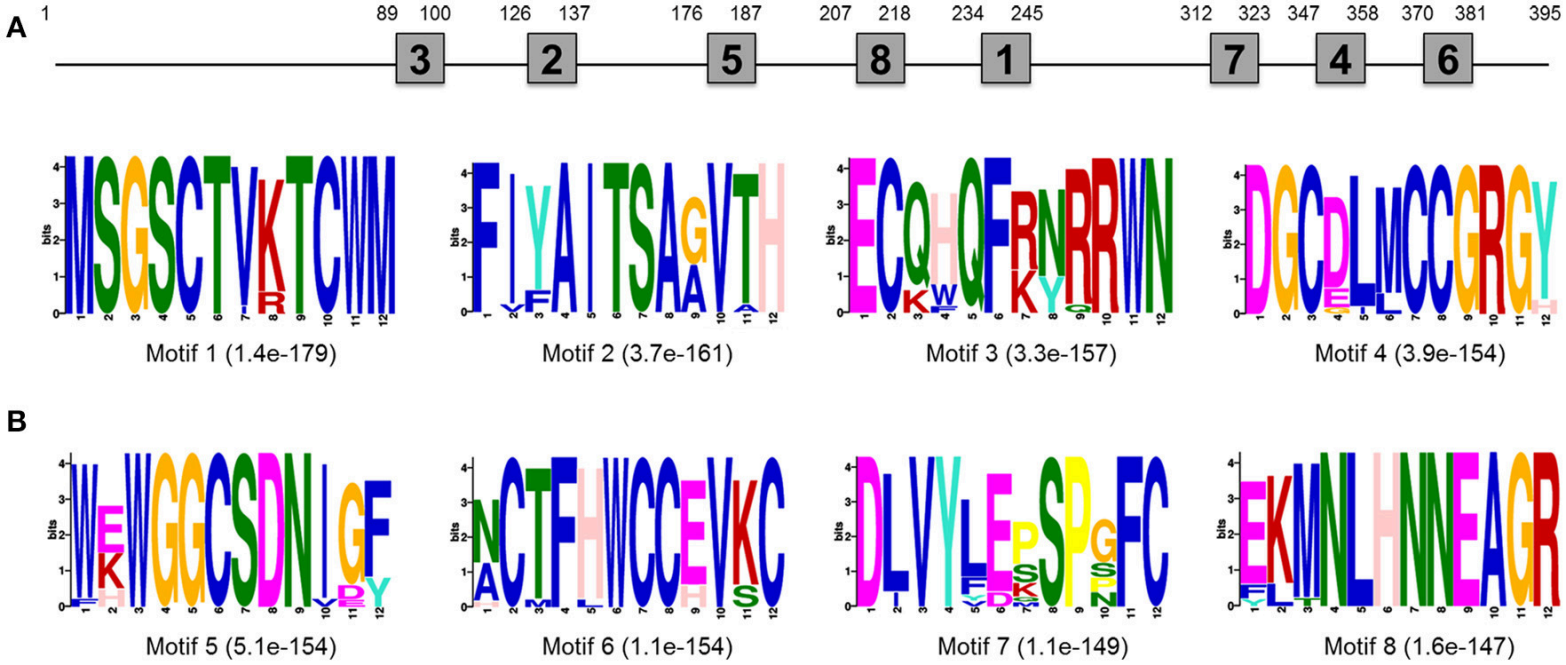
**Motif analysis of ***Wnt-1*** primary structure. (A)** Approximate location of each motif in the protein sequence. **(B)** The most conserved motifs. The number in the boxes corresponds to the numbered motifs. The number in parentheses represents the *e*-values.

**Figure 2:**
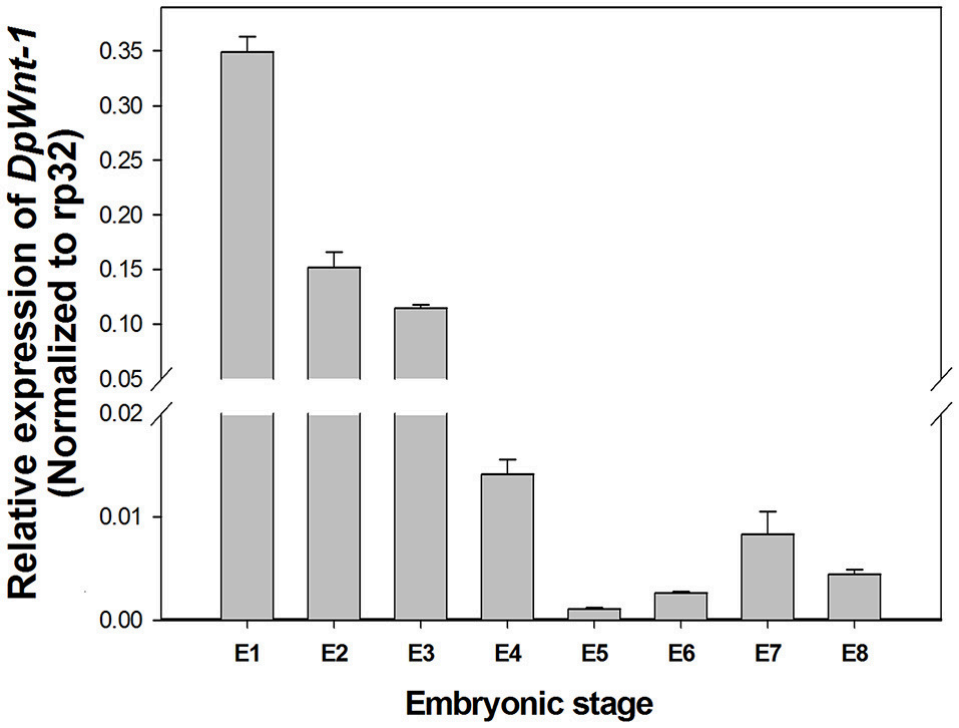
**Temporal expression of ***DpWnt-1***during embryonic stages**. The relative mRNA levels of *DpWnt-1* in embryos from day 1 to 8 (E1-8). *RP32* was used as a reference gene to normalize target gene expression. The data are presented as mean values ± S.E.M (*n* = 3).

EST sequence of *DpWnt-1* (GenBank accession #:KU640201) was initially obtained from *D. punctatus* transcriptome. The full length cDNAs of *DpWnt-1* contained 1182 nucleotides, which encodes 394 amino acids. The nucleotide sequence of *DpWnt-1* was rich in cysteine residues-a character of *Wnt* protein family (Figure S1). *Wnt-1* homologs from 18 species shared eight conserved motifs, which located between the N- and C-terminus (Figure 1). Phylogenetic relationship showed that DpWNT-1 clustered with other lepidopterans WNT-1 protein sequences (Figure S2). The expression of *DpWnt-1* peaked at the very beginning, declined during the development, and reached the minimum level at the end of embryogenesis (Figure 2), suggesting that *DpWnt-1* may play a vital role in *D. punctatus* during the early embryogenesis.

### CRISPR/Cas9 induced *DpWnt-1* mutations

To functionally characterize *DpWnt-1*, CRISPR/Cas9 mutagenesis system was introduced into *D. punctatus*. A total of 240 *D. punctatus* eggs were co-injected for each concentration of Cas9 mRNA and *DpWnt-1* sgRNAs, whereas 120 eggs were injected for the corresponding concentrations for the control EGFP sgRNAs (Table 2). Compared to co-injections of Cas9 protein and *DpWnt-1* guide RNAs with those targeting a control gene (EGFP), *D. punctatus* embryos with an inactive copy of *Wnt-1*showed a reduced hatching rate (22.5 and 30.5% at a concentration of 500 and 300 ng/μl, respectively), and a range of phenotypic effects (e.g., various body plan defects, absence of tissue differentiation). Among the 120 control eggs injected with EGFP sgRNAs/Cas9 mRNA, 57.5 and 64.2% individuals hatched at a concentration of 500 and 300 ng/μl, respectively. In comparison, 65.8% (79/120) wild type eggs hatched.

**Table 2:** **Embryonic mutagenesis induced by Cas9/sgRNA injection targeting ***DpWnt-1*****.

**Gene**	**sgRNA/Cas9 concentration (ng/μl)**	**Injected (n)**	**Phenotypic variation**				**Pupation (n)**
			**Defected segments (%)**	**Defected legs (%)**	**Malformed head (%)**	**Hatch rate (%)**	
*Wnt-1*	300/300	240	9.5	6.3	1.7	30.5	0
	500/500	240	22.9	7.5	2.5	22.5	1
EGFP	300/300	120	0	0	0	57.5	25
	500/500	120	0	0	0	64.2	37
WT	–	120	0	0	0	74.2	42

**Figure 3:**
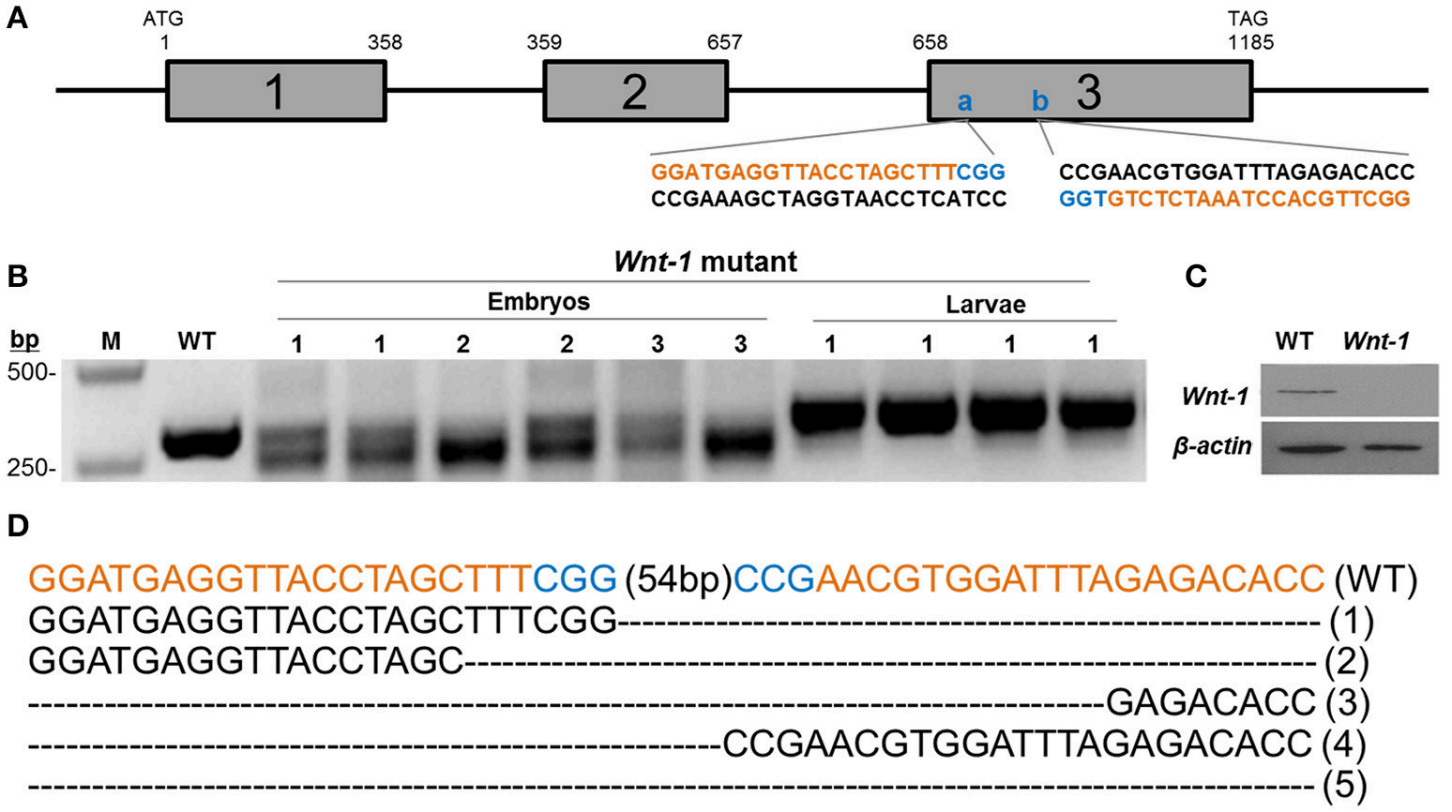
**Cas9/sgRNA-induced ***DpWnt-1*** mutations**. **(A)** Schematic representation of *Wnt-1* sgRNA targeting sites. The boxes indicate the three deduced exons of *DpWnt-1*, and the black line represents the untranslated regions and introns. The sgRNA targeting sites, **(A)** (74–96 bp) and **(B)** (151–173 bp), are located on exon 3. *Wnt-1-F* and *Wnt-1-R* were annealed to the upstream and downstream regions of the targeted site. **(B–D)** CRISPR/Cas9-induced mutagenesis of *DpWnt-1*. **(B)** Representative electrophoretogram of PCR products. Mutants with defective segments (1), defective legs (2), and malformed head (3) were sequenced. **(C)** *DpWnt-1* protein was undetectable in mutants by Western blotting analysis. **(D)** Various deletion genotypes. The fragment flanking the two targeted sites were deleted. The indel mutation genotype is noted on the right.

CRISPR/Cas9 system induced mutations in the pine moth with high efficiency. Eighty percentage (8 of 10) of the dissected embryos had mutations at the target sites, and the overall mutagenesis frequency was 32.9% in the injected generation at a higher dose (500 ng/μl). Similarly, at a lower dosage (300 ng/μl), 70% (7 of 10) of the dissected embryos had mutations at the target sites and the overall mutagenesis frequency was ~17.5% (Table 2). The genotypes of the wild types and *DpWnt-1* mutants were confirmed by both sequencing and Western blotting analysis (Figures 3B,C). All examined *DpWnt-1* mutants, including embryos and larvae, had alterations at the target sites that led to at least five type of deletions (Figure 3D). The deletion occurred at target sites individually, simultaneously, or was absent from both sites.

### Functional characterization of *DpWnt-1*

Knocking out *DpWnt-1* has great impact on eggs development. Most of eggs showed abdominal segments distortion and only some of them could hatch and develop into pupae, of which none reached the adult stage. When injected with 500 ng/μl of Cas9 mRNA and *DpWnt-1* sgRNA, 22.9% of the embryos showed abnormal anterior-posterior (A-P) axis and abdominal segmentation phenotypes, 7.5% showed defective legs, and 2.5% showed head malformations. In contrast, when the injection concentration is 300 ng/μl, 9.5% of embryos showed abnormal A-P axis and abdominal segmentation phenotypes, 6.3% showed defective legs, and 1.7% showed head malformations. As a control, 240 eggs were co-injected with EGFP-sgRNA/Cas9 mRNA. A total of 146 eggs (60.8%) hatched, and no morphological changes were observed (Table 2).

#### Patterning of the posterior segment from embryo to pupa

**Figure 4:**
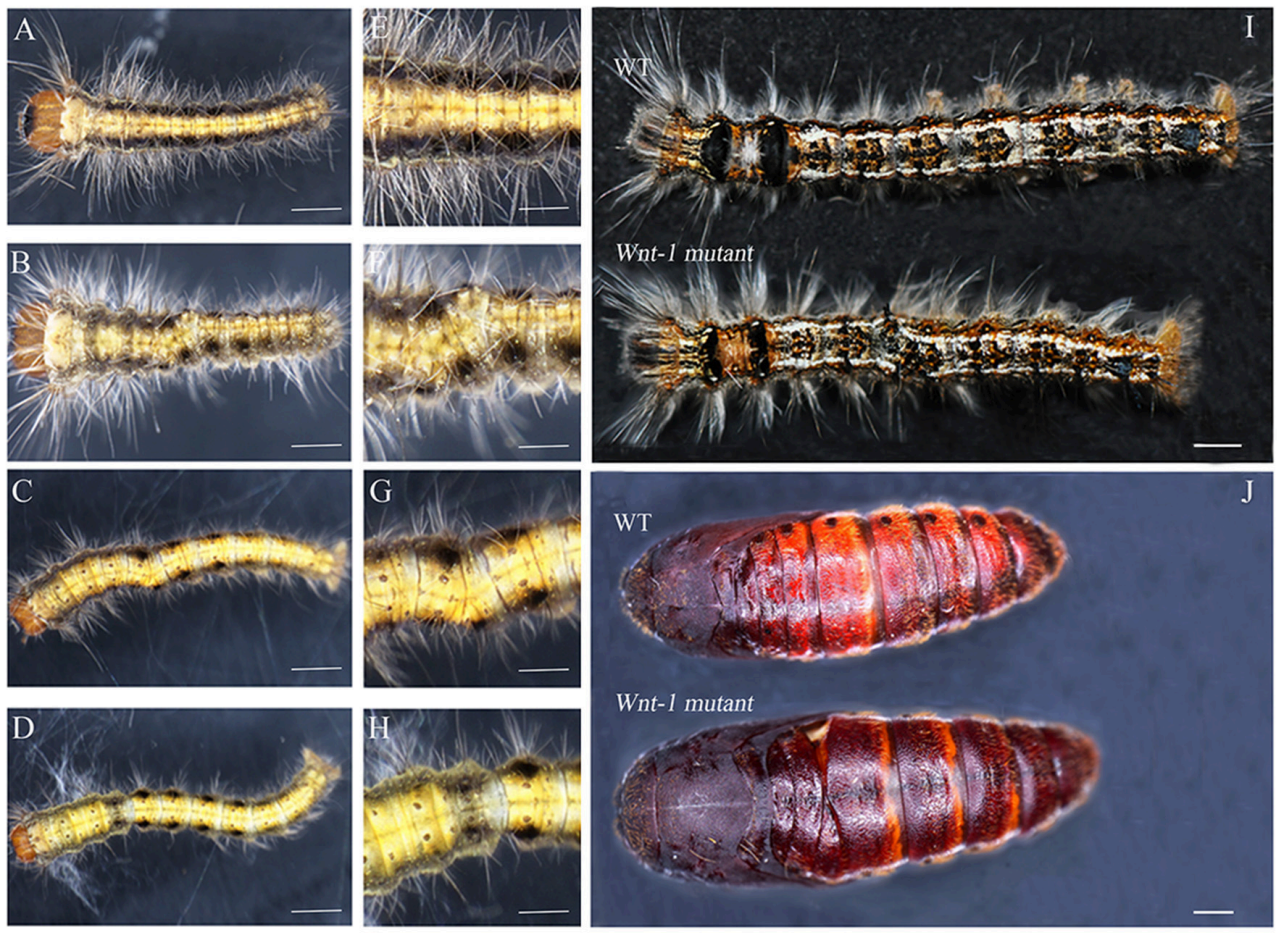
**Cas9/sgRNA-induced posterior segment defects in ***D. punctatus*** larvae and pupae. (A,E)** EGFP-specific sgRNAs/Cas9 mRNA control. **(B–D,F–H)** Mildly affected larvae resulting from *DpWnt-1* sgRNAs/Cas9 mRNA co-injection. Transformation of the abdominal segment from posterior to anterior. **(I)** Fifth instar larvae, wild type (up) and *DpWnt-1* mutant (down), displaying the transformation of A6/7 into A6. **(J)** Wild type and *DpWnt-1* mutant pupae. (**B,F)** The mutant larvae type I showed a transformation of A3/5 into A3 and a disturbance of the anterior-posterior axis. **(C,G)** The mutant larvae type II showed a transformation of A2/4 into A3 and a disturbance of the anterior-posterior axis. **(D,H)** The mutant larvae type III has extra pigmentation at A2. **(E–H)** Close-up images of the wild type and mutant individuals. The scale bars represent 0.5 mm **(A–D)**, 0.25 mm **(E–H)**, 50.0 mm **(I)**, and 2.0 mm **(J)**.

**Figure 5:**
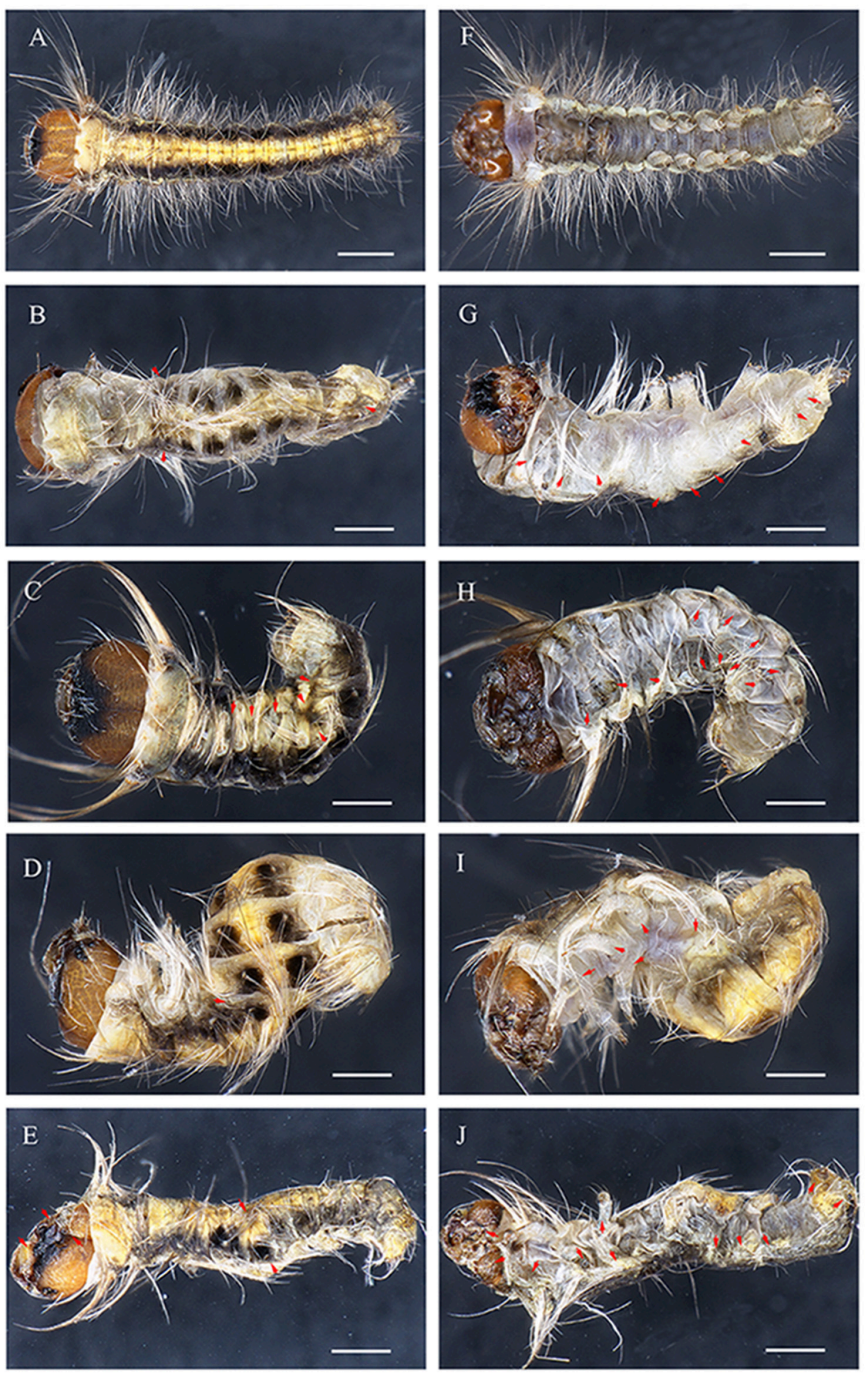
**Embryonic phenotypes in ***D. punctatus***. (A,F)** EGFP sgRNAs/Cas9 mRNA injected control embryo. **(B–E,G–J)** Severely affected embryo resulting from *DpWnt-1* sgRNAs/Cas9 mRNA injection. **(B,G)** Thoracic leg and prolegs missing on one side. **(C,H)** Compact body with thoracic legs and prolegs missing on both sides. **(D,I)** Twisted body without thoracic legs or patterning along anterior and posterior axis, with all prolegs missing. **(E,J)** Deformed body with malformed head, missing thoracic legs and prolegs on one side. All images were taken at the same magnification. Dorsal is on left and ventral is on right. The scale bars represent 1 mm.

**Figure 6:**
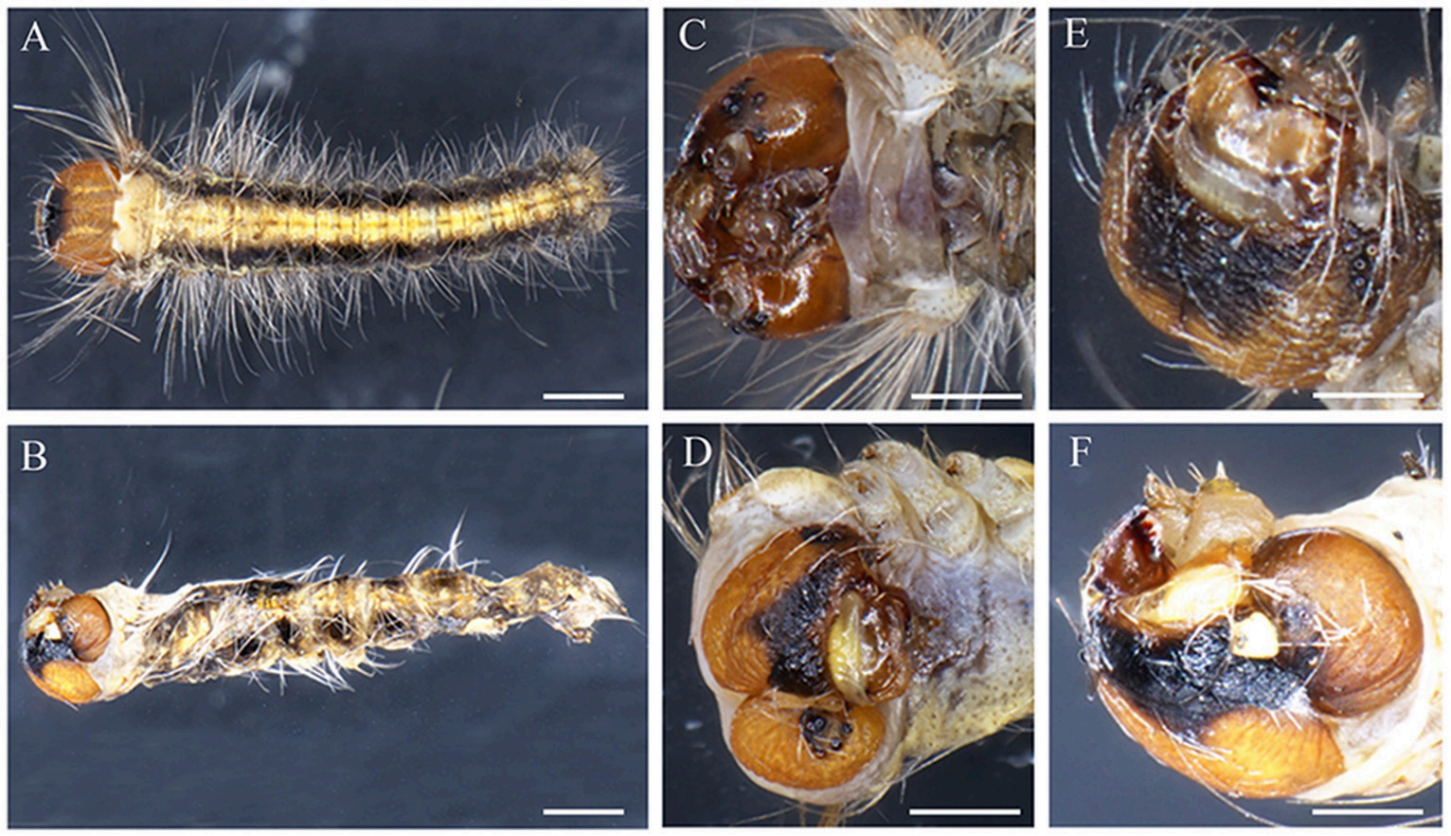
**Head phenotypes of ***DpWnt-1*** mutants. (A,C,E)** Wild type embryo. **(B,D,F)** Severely affected embryo with malformed head, missing thoracic legs and prolegs on both sides. The scale bars represent 1 mm.

*DpWnt-1* knockout led to visible abnormal abdominal formation phenotypes and abnormal patterning of the A-P axis (Figures 4–6). Some of the embryos showed the anteriorization of segments A2/7 (Figure 4). In some mutants, the loss of *DpWnt-1* led to the transformation of segments A2–A6 into more anterior abdominal segments (Figure 5). Some embryos showed a loss of epithelia on the dorsal side of the A3/5 segments, which was close to the intersegmental membrane and the dorsal mid line (Figures 4I,J). In other mutants, the boundaries between the abdominal segments and the anteroposterior body axis were discreet, as all of the abdominal segments (A2–A7) were fused together (Figure 5), indicating that *DpWnt-1* plays a role in posterior segmentation and A-P axis patterning. During the development, *DpWnt-1* mutants retained the posterior segment fusion and the truncated cuticle phenotypes and were unable to form posterior segments in a specific region (Figure 4).

#### Anterior body development

*DpWnt-1* signaling plays a crucial role in the development of the anterior segments in *D. punctatus*. *DpWnt-1* mutant larvae had missing appendages and displayed asymmetric anterior segment phenotypes (Figures 5, 6). In the wild type, the ecdysial line is localized in the middle of the head, and the lateral ocelli and antennae are located on both sides of the head (Figure 5A). In comparison with wild type larvae, partial lateral ocelli, antennae and intercalary were missing on the head of *DpWnt-1* mutants, while other mutants showed defective mouthparts with mandibular, maxillary and labial missing (Figures 5E,J, 6B–F).

#### Leg patterning

*DpWnt-1* is involved in the leg development, specifically on thoracic segments (T1–T3) and abdominal segments (A3–A6). The wild type embryo had three pairs of thoracic legs from the first to third thoracic segments and four pairs of prolegs from the third to sixth abdominal segments. In the type I mutant, some of the T1–T3 and A3–A6 segments were missing, and thoracic legs and prolegs were on one side of the segments (Figures 5B,G). In the type II mutant, some of the T1–T3 and A3–A6 segments were missing, and thoracic legs and prolegs were on both sides of the segments (Figures 5C,D,H,I). In the type III mutant, the legs on the T1–T3 thoracic segments did not follow the principle of symmetry and showed an asymmetrical distribution along the A-P axis. Moreover, the A3–A6 prolegs were missing on both sides of the segments (Figures 5E,J).

#### Pleiotropic impact of DpWnt-1 knockout

**Figure 7:**
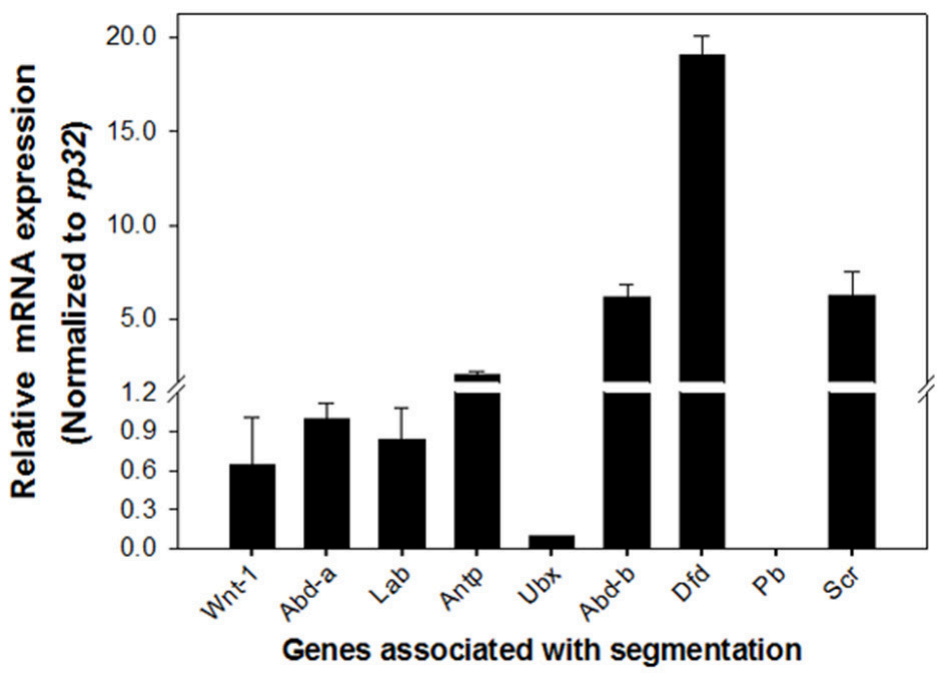
**Expression profiling in *DpWnt-1* mutants**. Compared to the controls, the mRNA expression of *Sex combs reduced* (*Scr*), *Deformed* (*Dfd*), and *Abdominal-b* (*Abd-b*) increased more than 4-fold in the *DpWnt-1* mutants. Others, including *Labial* (*Lab*), *Proboscipedia* (*Pb*), *Antennapedia* (*Antp*), *Ultrabithorax* (*Ubx*), and *Abdominal-a* (*Abd-a*), changed <2-fold. *Rp32* was used as reference gene for RT-PCR normalization. The data are presented as mean values ± S.E.M (*n* = 3).

The distinct phenotypes exhibited in *DpWnt-1* mutants suggested that *DpWnt-1* may participate in segmentation. *Hox* genes are known to be involved in segmentation. qRT-PCR analysis in 8-day old *DpWnt-1* mutant and wild type embryos results showed that *Sex combs reduced* (*Scr*), *Deformed* (*Dfd*), and *Abdominal-b* (*Abd-b*) were significantly upregulated while *Ultrabithorax* (*Ubx*) was downregulated in *DpWnt-1* mutants. The *DpWnt-1* mutants also showed slightly reduced expression levels of *Labial* (*Lab*), *Abdominal-a* (*Abd-a*), and *Antennapedia* (*Antp*), whereas *Proboscipedia* (*Pb*) was undetectable (Figure 7).

## Discussion

### Characteristics of *Wnt-1* homolog

Understanding the function of *Wnt-1* is critical for exploring its potential role in pest management. In this study, we cloned and characterized *DpWnt-1* homolog and identified one *Wnt-1* gene in *D. punctatus, DpWnt-1*. The motif and phylogenetic analyses confirmed that *DpWnt-1* is most closely related to *BmWnt-1* (Dhawan and Gopinathan, 2003).

In *Drosophila*, with long germ embryos, *Wnt-1* expression was first detected in the whole segments of the blastoderm during cellularization (Baker, 1987; Vorwald-Denholtz and De Robertis, 2011). In *Tribolium*, with short-germ embryos, *Wnt-1* was initially detected in the blastoderm stage, expressed sequentially from anterior to posterior with the germ band elongation and at the ventral portion of each segment during the late embryonic stage (Nagy and Carroll, 1994). In short/intermediate germ embryos, *Wnt-1* was detected in a broad median of the germ disk and then retracted posteriorly within segmentation process (Nakao, 2010). The expression pattern of *DpWnt-1* during the embryonic stage showed the same trend with that of *Bombyx* (Zhang et al., 2015). *BmWnt-1* was present in a maternal gradient and might play a role during the blastoderm formation (Nakao, 2010; Zhang et al., 2015). We hypothesized that *D. punctatus* may have a short/intermediate germ band, in which segmentation proceeds consecutively from anterior to posterior and show visible anterior and posterior segments after gastrulation.

### CRISPR/Cas 9 system in *D. punctatus*

In this study, embryonic injection of a mixture of sgRNAs/Cas9 mRNA successfully induced mutations in *DpWnt-1*, demonstrating that CRISPR/Cas9-mediated genome editing can specifically and efficiently induce gene alterations in *D. punctatus*. Besides *D. punctatus*, CRISPR/Cas9 system has also been exploited in seven other Lepidoptera species, including *B. mori, S. litura, S. littoralis, P. xylostella, P. xuthus, H. armigera*, and *D. plexippus*, to manipulate genes associated with development (embryogenesis), pigmentation, metamorphosis, resistance mechanism, and adult mating (Wang et al., 2013, 2016; Li et al., 2015; Bi et al., 2016; Huang et al., 2016; Koutroumpa et al., 2016; Markert et al., 2016; Zhu et al., 2016). Moreover, the frequency of mutation is dose dependent. Knocking out *DpWnt-1* led to a high embryonic mortality (~70%), and none of the *DpWnt-1* mutants could developed from larva to adult, suggesting that *DpWnt-1* is a potential candidate for conditional lethal gene.

Although CRISPR/Cas9 system is clearly applicable in *Dendrolimus*, additional experiments are needed to fully established this genome editing technology in this major forest pest. *In situ* hybridization study of *DpWnt-1* not only will validate genome editing results at the translational level, but also provide the spatial expression pattern, and the potential *Hox* targets. Also, without genome information, we could not pinpoint the off-target effects, which is a routine problem for this technology. With other genomic resources (Yang et al., 2016), the potential off-target effects can be predicted.

### Involvement of *DpWnt-1* in segmentation and appendage development

#### DpWnt-1 in posterior segmentation

*Wnt-1* has been documented to play an important role in A-P axis patterning and segment development during embryogenesis. In *DpWnt-1* mutants, abnormal posterior segments from Abdomen 2 (A2) to Abdomen 7 (A7) were observed along with affected A-P axis patterning. An examination of *Hox* genes in *Wnt-1* mutants suggested that *DpWnt-1* may have a connection with *Hox* genes in regulating insect segmentation. Our results for the function of *DpWnt-1* are consistent with those of *Bombyx*, in which *DpWnt-1* plays a role in body segmentation. However, *Wnt-1* appears to have a different effect on the expression of other genes, as all *Hox* genes were significantly down-regulated in *Bombyx* (Zhang et al., 2015). Consistent with *Drosophila, Wingless* signaling ensures the formation of the posterior segment boundaries (Larsen et al., 2003). However, depletion of *Wnt-1* in *G. bimaculatus, Oncopeltus fasiatus*, and *Tribolium*, does not reduce the number of segments, but depletion of other *Wnt* signaling genes like *GbArm* leads to abdominal segments defects in embryos, removal of *OfPan* results in truncates segmentation, depleting of *TcWnt-8* brings about embryos lacking abdominal segments and additional removal of *TcWnt-1* enhances this phenotype (Miyawaki et al., 2004; Angelini and Kaufman, 2005; Shah et al., 2011). All of these results indicate that *DpWnt-1* plays a role in segmentation in *D. punctatus*.

#### DpWnt-1 in anterior segmentation

The genetic regulation of the anterior development in insects is poorly understood. According to Rogers and Kaufman (1996), head was divided into three cephalic segments (ocular, antennal, and intercalary) and three gnathal segments (mandibular, maxillary, and labial). In animals, *Wnt-1* is involved in the head development, including eyes, mesencephalon and metencephalon (Bally-Cuif et al., 1995; Friedrich, 2003; Lekven et al., 2003; Rossi et al., 2007). In *D. melanogaster*, temporal regulation of *Wnt* signaling is critical for the differentiation of antennal and maxillary organs (Lebreton et al., 2008). In *Tribolium, Wnt/*β*-catenin* signaling is required for the anterior development, which is needed for head patterning after cellularization (Bolognesi et al., 2008; Fu et al., 2012; Benton et al., 2013). Consistent with previous observations, both anterior and posterior sequential segmentation were affected in *DpWnt-1* mutants. Besides, partial cephalic segments and gnathal segments of the mutants were missing or defected. These results support the hypothesis that *Wnt* signaling is an integral part of an ancestral metazoan mechanism that specify the architecture of posterior and anterior segments.

#### DpWnt-1 in appendage development

The morphological plasticity of appendages represents a crucial aspect of animal body plan. Knocking out *DpWnt-1* produced defects in appendage development. No discernible defects in the appendages were found in mildly affected individuals (Figure 5). In severely affected individuals, however, lateral ocelli, antennae, the thoracic legs and prolegs were missing (Figure 6). Among these mutants, some thoracic legs or prolegs were distributed asymmetrically along the normal AP axis (Figure 6), suggesting that the specification of appendages in *Dendrolimus* requires *DpWnt-1*. Some of the defects, such as the loss of prolegs could be the indirect consequences of segmentation defects. Consistent with other holometabolous taxa, including Coleoptera, Lepidoptera, Hymenoptera and Diptera, *Wnt-1* signaling is involved in post-embryonic appendage development (Bejsovec and Peifer, 1992; Siegfried et al., 1994; Sato et al., 2008; Shah et al., 2011; Zhang et al., 2015). This is different from taxa that undergo incomplete metamorphosis, of which appendage development requires *Wnt-1* to interact with other genes, such as in *G. bimaculatus* (Miyawaki et al., 2004). Although *Gbwg* knockouts by RNAi showed no significant impacts on segmentation, *GbWnt/GbArm* signaling was involved in the posterior sequential segmentation during embryogenesis. In *P. americana, Wnt* signaling engaged in cross talk with *caudal* and *Notch* signaling in the regulation of growth and segmentation (Chesebro et al., 2013). In *O. fasiatus, Wnt* signaling played a role in body segmentation but not in appendage development (Angelini and Kaufman, 2005). Based on these results, we propose that the function of *Wnt* signaling is conserved among insects even though *Wnt-1* gene has diverse functions in different species.

In summary, our study demonstrates that genome editing using CRISPR/Cas9 system is feasible in *Dendrolimus*. This provides a brand new tool for conducting functional genomic research in a major forest pest. Furthermore, the results from the functional characterization of *DpWnt-1* demonstrated that this gene could potentially be utilized as a specific lethal gene in RIDL. CRISPR/Cas9 system could also be used to create transgenic lines to screen for dominant suppressors driven by specific promoters to provide candidate genes for the control of *Dendrolimus*.

## Author contributions

HL designed and conceived the study. XZ, HL analyzed the data. HL, XZ, YH, ZZ, and QL wrote the manuscript. All authors approved the final version of the manuscript.

### Conflict of interest statement

The authors declare that the research was conducted in the absence of any commercial or financial relationships that could be construed as a potential conflict of interest.
