# Supplementary material for: Genome Editing of Wnt-1, a Gene Associated with Segmentation, via CRISPR/Cas9 in the Pine Caterpillar Moth, Dendrolimus punctatus
Source: Front Physiol. 2017 Jan 6;7:666. doi: 10.3389/fphys.2016.00666 (PMC5216022; doi:10.3389/fphys.2016.00666)
Supplement: Supplementary file 1 [file DataSheet1.docx]

**Supplementary Materials**


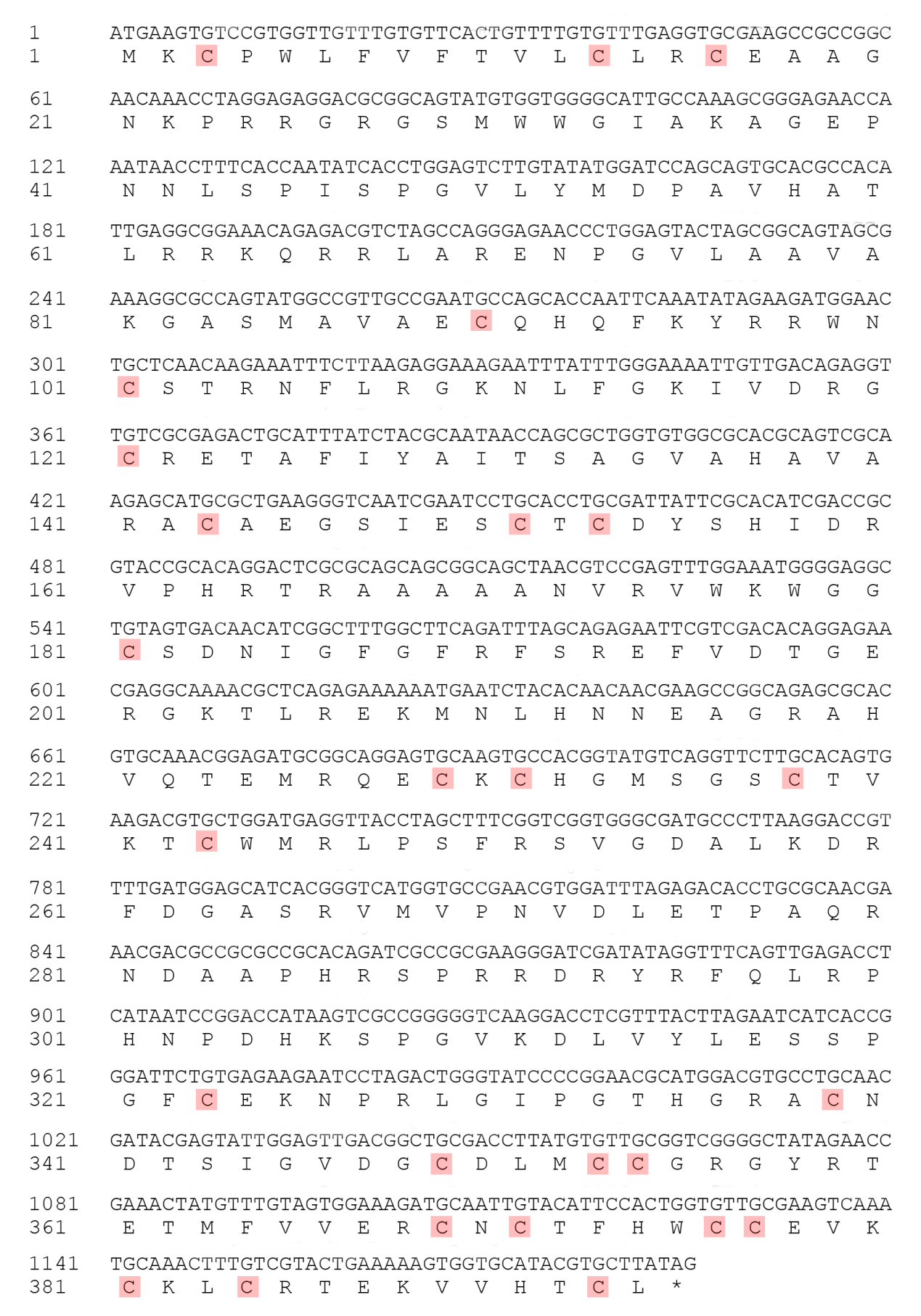


**Figure S1 *DpWnt-1* nucleotide sequence.** Cysteine residues were highlighted in red.


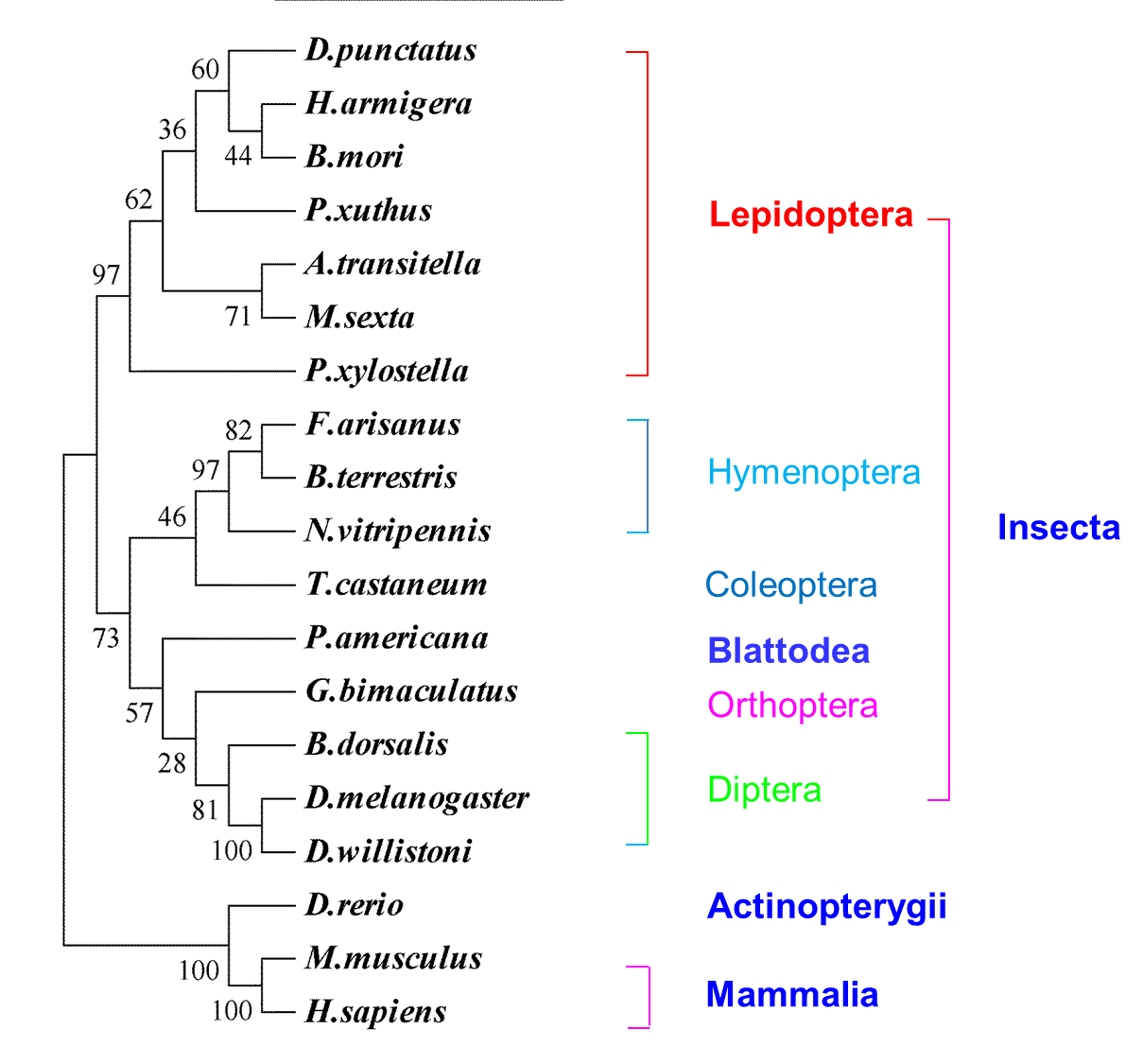


**Figure S2 Phylogenetic relationship of *DpWnt-1* with homologues from other species.** Phylogenetic relationships of 18 *Wnt-1* homologues were resolved by hierarchical cluster analysis based on their ORFs. Species included in this analysis were *B. dorsalis, B. terrestris, B. mori, P. xuthus, P. americana, N. vitripennis, H. armigera, F. arisanus, D. willistoni, D. melanogaster, G. bimaculatus, A. transitella, P. xylostella, M. sexta, D. rerio, H. sapiens, M. musculus* and *T. castaneum.* The number above or below the branches indicated bootstrap values with 1000 replications.


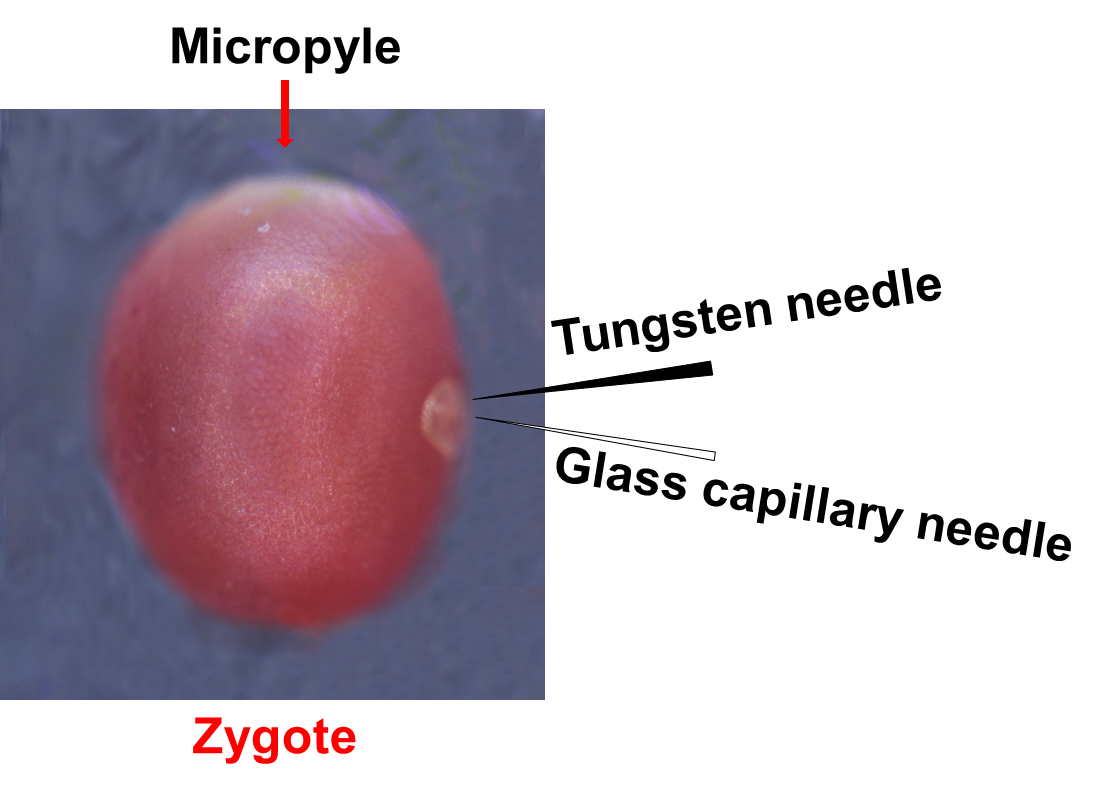


**Figure S3 Double needle injection system for *D. punctatus* eggs.** The micropyle is lining up on top.
